# Amyloid β‐induced cardiac neurotrophic signaling disruption drives heart dysfunction in Alzheimer's disease

**DOI:** 10.1002/alz70855_106732

**Published:** 2025-12-24

**Authors:** Andrea Elia, Rebecca M Parodi Rullan, Rafael Vazquez‐Torres, Ashley M Carey, Silvia Fossati

**Affiliations:** ^1^ Lewis Katz School of Medicine, Temple University, Philadelphia, PA, USA; ^2^ Alzheimer's Center at Lewis Katz School of Medicine, Temple University, Philadelphia, PA, USA; ^3^ Alzheimer's Center Lewis Katz School of Medicine, Temple University, Philadelphia, PA, USA; ^4^ Alzheimer's Center at Temple, Lewis Katz School of Medicine, Philadelphia, PA, USA

## Abstract

**Background:**

Alzheimer's disease (AD) profoundly disrupts neurotrophic factors (NTFs) signaling in the brain, including key players like NGF and BDNF, which are essential for maintaining healthy neuronal homeostasis. A loss of NTFs may also instigate peripheral nervous system dysfunction, potentially predisposing AD patients to develop cardiovascular disorders of different etiology. Despite the growing recognition of cardiac abnormalities in AD, the mechanisms through which AD pathology impairs the brain‐heart axis and affects myocardial innervation and function have remained poorly understood.

**Methods:**

This study comprehensively analyzes cardiac physiology, amyloid pathology, neurotrophic factor depletion, and cardiac neuronal fiber degeneration in Tg2576‐AD mice, human cardiomyocytes, and human AD postmortem left ventricular (LV) heart tissue.

**Results:**

Our results demonstrate that AD pathology leads to increased myocardial fibrosis, amyloid β (Aβ) deposition, and significant remodeling of the brain‐heart axis neuro‐signaling pathway, culminating in myocardial denervation and impaired cardiac function. Notably, Aβ oligomers were found to reduce BDNF expression in human cardiomyocytes by disrupting CREB function, the key transcription factor for BDNF expression. Postmortem analysis of human LV tissue from AD patients confirmed the animal and cell findings.

**Conclusion:**

Collectively, our research unveils a previously unrecognized mechanism by which Aβ dysregulates cardiac neurotrophic signaling, emphasizing the relevance of cardiac degeneration in AD. Our findings highlight the importance of addressing cardiac complications in AD management, thus opening novel translational avenues for future precision medicine investigations that may help to reevaluate the clinical approaches to AD therapy.

